# The appearance of “faggot Auer rods” in blasts of non-M3 acute myeloid leukemia

**DOI:** 10.3389/fonc.2026.1791126

**Published:** 2026-06-08

**Authors:** Cui Zhang, Jia Li, Wenfang Zhuang

**Affiliations:** Medical Laboratory, Shidong Hospital Affiliated to University of Shanghai for Science and Technology, Shanghai, China

**Keywords:** acute myeloid leukemia, acute promyelocytic leukemia, Auer rods, faggot-like, fusion gene

## Abstract

**Background:**

Acute myeloid leukemia (AML) is an acute leukemia characterized by aberrant proliferation of myeloid blasts in the bone marrow. Auer bodies are identifiable in the blasts of certain AML subtypes, but their “faggot-like” aggregation is uncommon, associated with strong peroxidase positivity, and readily misidentified as abnormal promyelocytes.

**Case presentation:**

A 79-year-old male was admitted for leukocytosis detected on physical examination. One month prior, he had marked leukocytosis (unspecified value) with predominant monocyte elevation. Admission complete blood count (CBC) confirmed leukocytosis and elevated monocyte percentage. Peripheral blood (PB) smear demonstrated 35% abnormal leukocytes, and bone marrow (BM) aspirate revealed these cells accounting for 40%. Morphologically, the cells had round nuclei and abundant cytoplasmic granules, with most of the cytoplasm filled with “faggot-like” Auer bodies (strongly positive for peroxidase). Flow cytometry revealed blasts positive for CD117, CD33, and MPO, with minimal positivity for CD13. Further investigations (leukemia fusion gene detection, FISH, chromosome Analysis, next-generation sequencing, and whole-transcriptome sequencing) failed to detect any APL-specific fusion genes, and the patient was ultimately diagnosed with non-M3 subtype AML.

**Conclusion:**

This case is a special type of acute myeloid leukemia (AML) with morphological features highly resembling acute promyelocytic leukemia (APL) but without the associated fusion gene. Faggot-like Auer bodies, once considered a hallmark of APL, are not pathognomonic for APL, and diagnosis based solely on morphology is susceptible to misdiagnosis. This report aims to accumulate clinical experience, provide a reference for the diagnosis and management of similar cases, and reduce the incidence of misdiagnosis and missed diagnosis.

## Introduction

Acute Myeloid Leukemia (AML) is a highly heterogeneous hematological malignancy originating from myeloid hematopoietic stem/progenitor cells. Its core pathological feature is the clonal abnormal proliferation and differentiation arrest of myeloid blasts, which leads to suppression of normal hematopoiesis. Accurate classification is the core prerequisite for individualized treatment and prognostic assessm (1). Acute Promyelocytic Leukemia (APL) is an independent subtype of AML with unique clinical characteristics, accounting for approximately 10% of all AML cases (2). Morphologically, the vast majority of APL patients exhibit increased abnormal promyelocytes in the bone marrow and peripheral blood, with dense azurophilic granules in the cytoplasm. “Faggot-like” Auer rods can be detected in about 90% of cases, serving as initial screening clues rather than definitive diagnostic criteria. The gold standard for APL diagnosis is the detection of -RARα-related fusion genes, among which the PML-RARα fusion gene resulting from t (15;17) translocation is the most common, and detection of these fusion genes is key to precise treatment (3). Clinically, cases with morphologically suspected APL but ultimately non-APL are rare. This article reports a challenging case: the patient was treated with all-trans retinoic acid but showed poor therapeutic response, and no RARα-related fusion genes were detected by flow cytometry (FCM), reverse transcription-polymerase chain reaction (RT-PCR), and RNA sequencing (RNA-seq), leading to a final diagnosis of non-M3 type AML. This study explores the diagnostic difficulties and misdiagnosis points of AML through this case, emphasizes the importance of RARα-related fusion gene detection, and provides reference for the standardized diagnosis and treatment of difficult AML cases.

## Case presentation

A 79-year-old male patient was incidentally found to have persistent leukocytosis with monocytosis during routine physical examinations over one month. The patient had a decades-long history of leukopenia and anemia, which had previously responded to symptomatic treatment with transient resolution of anemia. Routine tests in July 2024 showed normal white blood cells (WBC) count, moderate anemia, and elevated monocyte proportion (WBC:3.9×10^9^/L; hemoglobin:60g/L; monocytes:34%). The patient did not pursue further evaluation at that time and continued symptomatic management. By October 2024, follow-up testing demonstrated significant leukocytosis, prompting referral to our hematology department for evaluation. To further clarify the cause of leukocytosis, the patient was admitted to the Hematology Department of our hospital on October 31, 2024. Physical examination revealed:marked pallor consistent with severe anemia, no palpable superficial lymphadenopathy, absence of hepatosplenomegaly, no cutaneous or mucosal jaundice, petechiae, or ecchymoses.

Laboratory tests showed a white blood cell count of 25.67×10^9^/L, red blood cell count of 0.85×10^12^/L, hemoglobin of 29 g/L, platelet count of 92×10^9^/L, and an increased monocyte proportion of 28.4% in the classification count. Other relevant auxiliary examinations: Folic acid, Vitamin B12, and solid tumor-related markers were all within normal ranges. Peripheral blood smear morphology was urgently performed, and a bone marrow aspiration was conducted to evaluate hematopoiesis.

## Methods

Peripheral blood and bone marrow smears were prepared using Wright-Giemsa staining and examined under an Olympus-CX43 optical microscope (Japan). Bone marrow specimens underwent cytochemical staining for peroxidase (POX) and naphthol AS-D chloroacetate esterase. Immunophenotyping analysis and detection were performed by flow cytometry; detection of leukemia fusion genes by reverse transcription polymerase chain reaction(RT-PCR); the RARα gene rearrangement was detected by fluorescence *in situ* hybridization(FISH) technology; cytogenetic analysis of chromosome karyotypes and next-generation sequencing(NGS) methods for the detection of myeloid tumor-related genes; the whole transcriptome sequencing of hematologic malignancies was used to detect special fusion or mutation genes in bone marrow (All special tests related to immunophenotype, cytogenetics and molecular biology were conducted by Kingmed Diagnostics Group Co., Ltd., Shanghai, China). The studies involving humans were approved by the ethics committee of Shidong Hospital Affiliated to University of Shanghai for Science and Technology.

## Results

**Figure 1:**
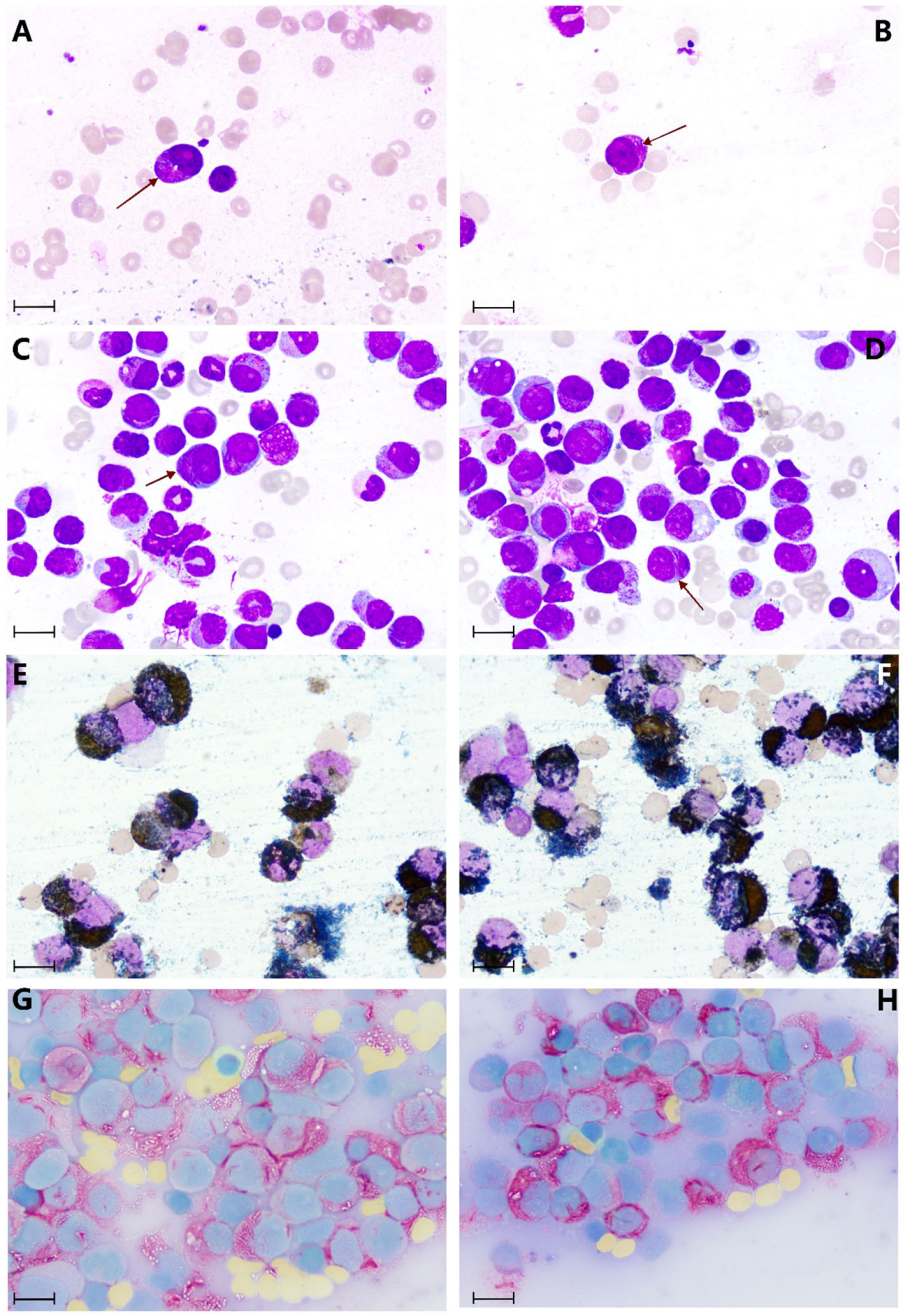
Results of cytomorphology and cytochemical staining of peripheral blood smears and bone marrow smear **(A, B)** Abnormal leukocyte morphology in peripheral blood smear (Wright-Giemsa stain; magnification: ×1000). **(C, D)** Abnormal leukocyte morphology in bone marrow smear (Wright-Giemsa stain; magnification: ×1000). **(E, F)** Bone marrow smear showing strongly positive peroxidase (POX) staining in abnormal leukocytes (peroxidase stain; magnification: ×1000); **(G, H)** Bone marrow smear showing positive or strongly positive naphthol AS-D chloroacetate esterase staining in abnormal leukocytes (naphthol AS-D chloroacetate esterase stain; magnification: ×1000). The cell indicated by the red arrow is a cell containing “faggot-like” Auer bodies. Scale bar 20 μm.

**Figure 2:**
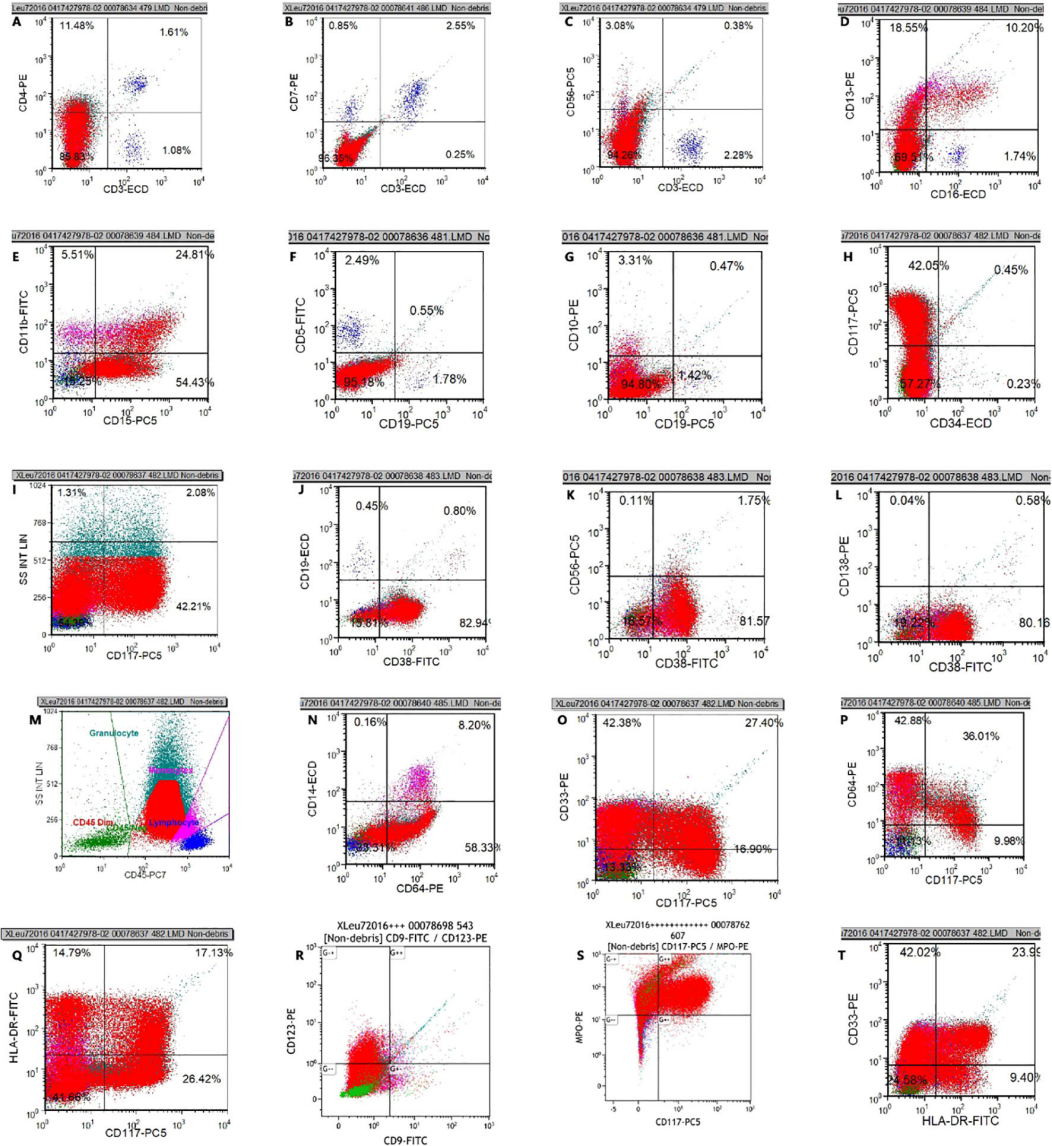
Flow cytometric immunophenotyping results of bone marrow cells showed that 42.2% of the CD117+ cells exhibited the following immunophenotype: CD45dim+, CD117+, CD33+, CD64+, CD38+, CD15+, intracellular MPO+, partial HLA-DR+, partial CD123+, a small amount of CD13+, CD9-, CD34-, CD10-, CD14-, CD56-, CD4-, CD7-, CD19-, CD3-. The immunophenotype is consistent with acute myeloid leukemia (AML). **(A–C)** CD3-, CD7-, CD56-; **(D)** a small amount of CD13+; **(E)** CD15+; **(F, G)** CD19-, CD10-; **(H, I)** CD117+ (42.2%), CD34-; **(J–L)** CD38+, CD56-; **(M)** CD45dim+;**(N)** CD14-, CD64+; **(O)** dual positivity for CD117 and CD33 (27.4%); **(P)** dual positivity for CD117 and CD64 (36.01%); **(Q)** partial HLA-DR+; **(R)** CD9-, partial CD123+; **(S)** intracellular MPO+; **(T)** dual positivity for CD33 and HLA-DR (23.9%).

Both bone marrow and peripheral blood smears revealed numerous of proliferating abnormal leukocytes, accounting for 35% in peripheral blood and 40% in the bone marrow. These cells displayed medium-sized, round to oval morphology with abundant cytoplasm containing variable amounts of purplish-red azurophilic granules. Notably, most cells exhibited characteristic “faggot-like” Auer rods. The nuclei appeared round or oval with fine chromatin and distinct nucleoli (**Figures 1A–D**). Bone marrow smear peroxidase(POX)staining showed:100% strong positivity in abnormal white blood cells, while AS-D naphthol esterase staining showed:100% positivity in abnormal white blood cells, with partial showing strong positivity (**Figures 1E–H**). The immunophenotype of bone marrow cell flow cytometry showed that a group of cells expressed CD45dim+, CD117+, CD33+, CD64+, CD38+, CD15+, intracellular MPO+, partial HLA-DR+, partial CD123+, a small amount of CD13+, and negative for CD9, CD34, CD10, CD14, CD56, CD4, CD7, CD19, and CD3 (**Figure 2**), their proportions account for 42.2% of the total number of nuclear cells, and the immunophenotypes consistent with AML.

**Figure 3:**
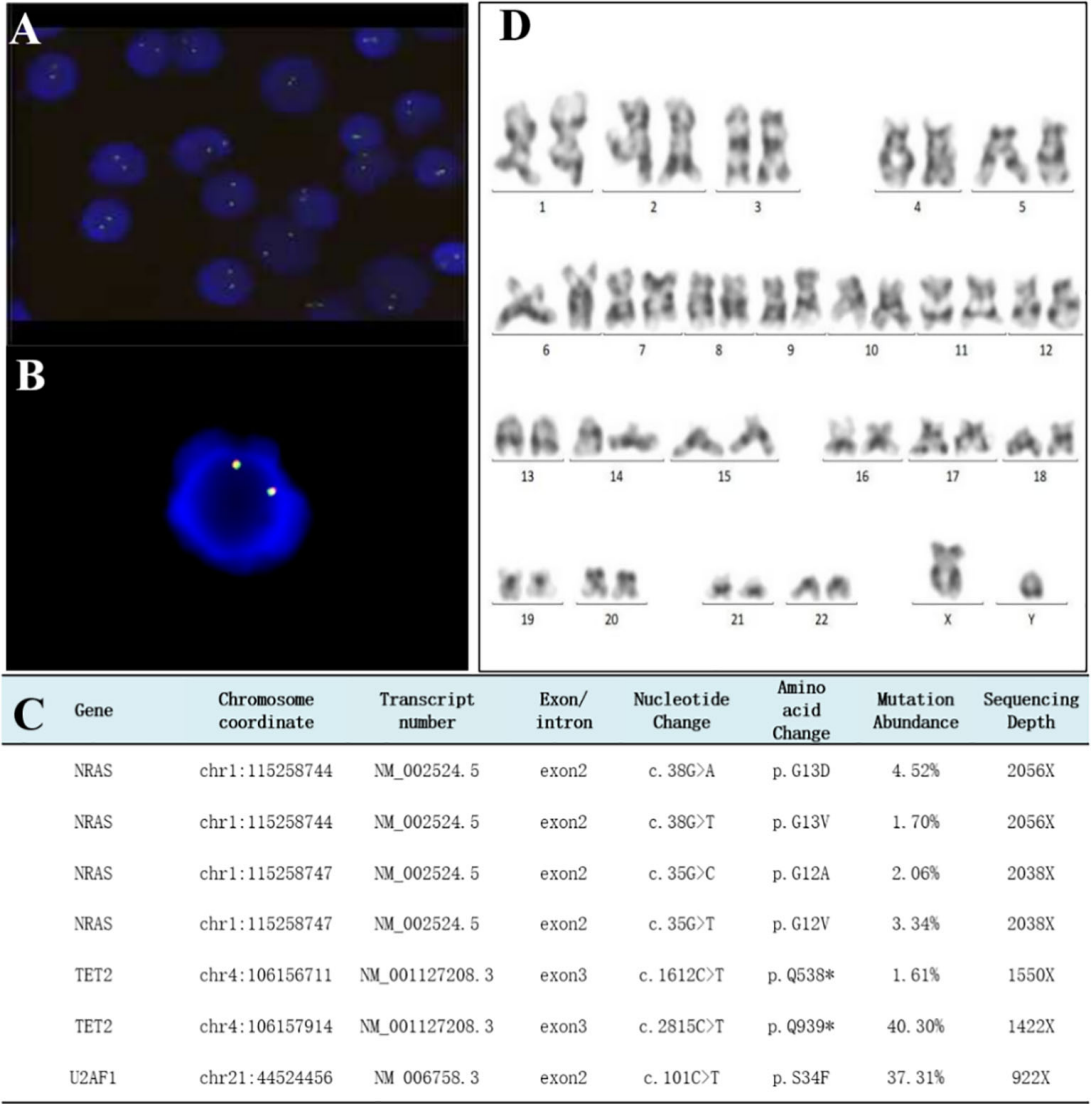
Results of fluorescence *in situ* hybridization, next-generation sequencing, and cytogenetic testing in bone marrow cells: **(A, B)** Fluorescence *in situ* hybridization(FISH) testing of bone marrow cells was negative. **(C)** Next-generation sequencing(NGS)of bone marrow cells detected the following myeloid gene mutations: NRAS (p. G13D 4.52%), NRAS (p. G13V 1.70%), NRAS (p. G12A 2.06%), NRAS (p. G12V 3.34%), TET2 (p. Q538* 1.61%), TET2 (p. Q939* 40.30%), U2AF1 (p. S34F 37.31%). **(D)** Cytogenetic testing of bone marrow cells revealed a normal karyotype: 46, XY.

Reverse transcription polymerase chain reaction(RT-PCR) testing of bone marrow aspiration cell for 43 common leukemia fusion genes (including *PRKAR1A::RARα*, *NUMA1::RARα*, *ETV6::PDGFRA*, *FIP1L1::RARα*, *STABT5B::RARα*, *PML::RARα*, *BCR::ABL1*, *RUNX1::RUNX1T1 (AML1::ETO)*, *CBFβ::MYH11*, *ZBTB16:RARα (PLZF::RARα)*, *FIP1L1::PDFFRα, NPM::RARα*, etc.) and fluorescence *in situ* hybridization for RARα gene rearrangement both showed negative results for fusion genes and rearrangements (**Figures 3A, B**). Next-generation sequencing(NGS)of myeloid tumor-related genes in bone marrow cells detected: NRAS gene p.G13D, p.G13V, p.G12A, p.G12V mutations; TET2 gene p.Q538*, p.Q939* mutations; and U2AF1 gene p.S34F mutation (**Figure 3C**). Cytogenetic analysis of bone marrow cells showed 17 metaphase chromosomes, revealing a normal karyotype: 46, XY (**Figure 3D**).

**Figure 4:**
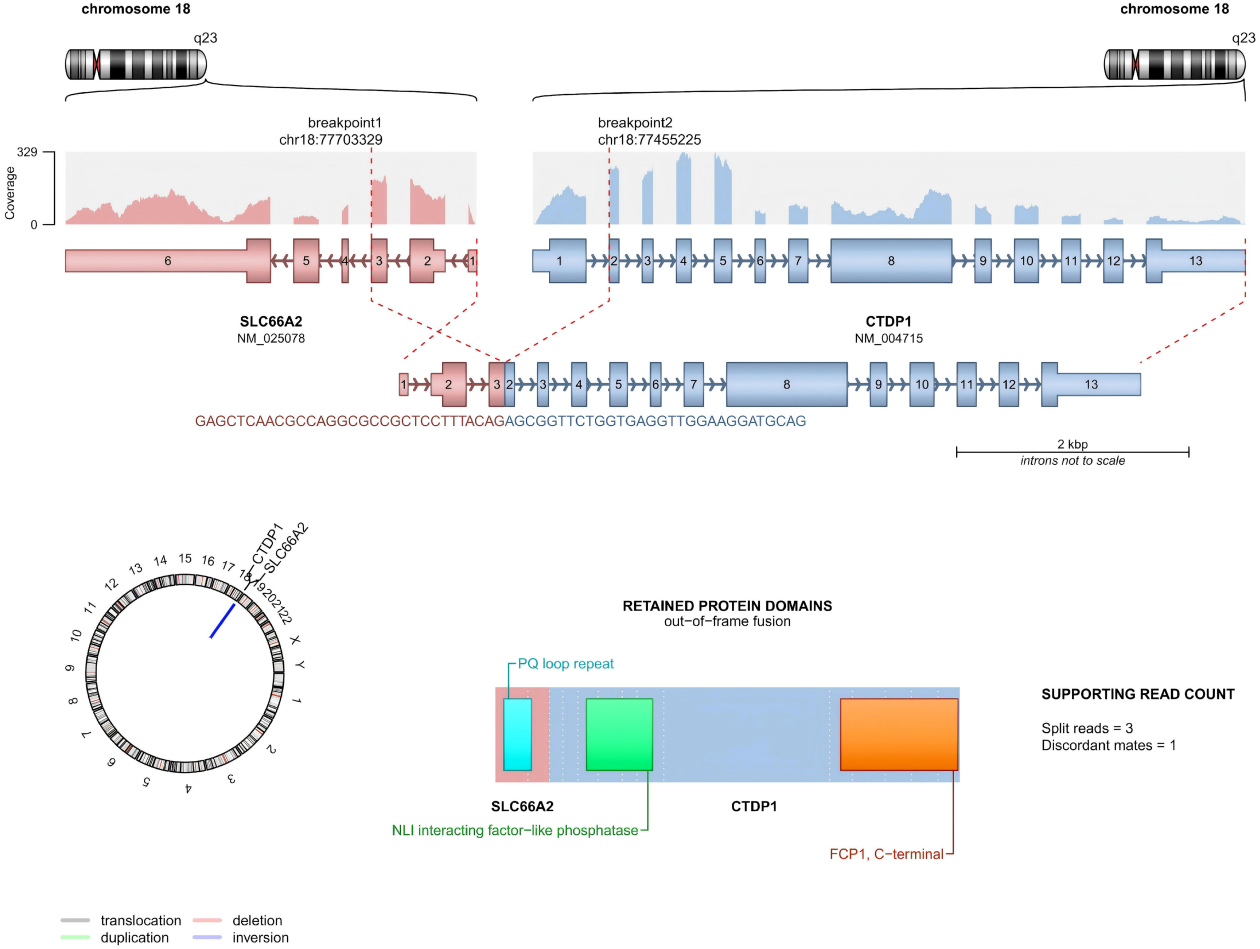
Whole transcriptome sequencing (RNA-seq) results of hematologic malignancies: The *SLC66A*2*::CTDP1* gene fusion was detected, which occurs in the q23 region of chromosome 18. This fusion gene has been sporadically detected in B-ALL, and its current clinical significance remains unclear.

Since abnormal cells resembling promyelocytes were observed in both bone marrow and peripheral blood smears, the morphological oral report suggested ruling out the possibility of “acute promyelocytic leukemia,” and the patient was prophylactically treated with all-trans retinoic acid (ATRA). However, when the fluorescence *in situ* hybridization (FISH) results, fusion gene analysis, and myeloid tumor gene mutation results were successively obtained, the possibility of typical APL was ruled out. The clinicians then diagnosed the patient with “acute myeloid leukemia (non-M3 type)”, and the patient received first-line therapy with azacitidine (100mg/m²/day × 7 days) plus venetoclax (200mg daily), with dose adjustments based on laboratory monitoring (4). Meanwhile, in order to further understand whether there are other rare types of RARα fusions, hematological tumor whole transcriptome sequencing (RNA-seq) was performed with the patient’s consent. The results showed no rare RARα fusions but detected an uncommon fusion gene classified as category III: *SLC66A2::CTDP1* gene fusion (**Figure 4**).

Following a comprehensive MICM evaluation, the diagnosis of acute myeloid leukemia (non-M3 type) was confirmed. After completing two cycles of chemotherapy according to the AML (non-M3) treatment protocol, a bone marrow re-examination on January 13, 2025 showed no detectable blasts by morphology or flow cytometry, indicating interim remission. After discharge, the patient continued oral venetoclax (1 tablet once daily) until discontinuation on February 3, 2025. On February 6, 2025, the patient was re-admitted for chemotherapy, and bone marrow examination showed a resurgence of blasts at 60.5%, exceeding the initial presentation level. Given the increased proportion of promyelocytes observed in both bone marrow smear and flow cytometry results, the chemotherapy regimen was adjusted to azacitidine 100mg/day (days1–7), venetoclax 200mg once daily, homoharringtonine 1mg/day (days1–3), Upon completing the treatment course, the patient was discharged and maintained on oral venetoclax. A comparison of blood cell counts and coagulation indices before and after chemotherapy is presented in **Table 1**. Two days after discharge, the patient was readmitted due to a respiratory tract infection and, unfortunately, passed away on March 4, 2025, due to viral pneumonia complicated by septic shock and respiratory failure. The survival period was just over four months.

**Table 1:** The patient’s blood cells counts and conglation indexs at different stages.

	1st cycle of therapy		2nd cycle of therapy		3rd cycle of therapy		4th cycle of therapy		Normal range
	before	after	before	after	before	after	before	after (death)	
white blood cells (×109/L)	25.67	1.04	1.29	1.71	1.45	2.54	21.26	1.18	3.50-9.50
red blood cells (×1012/L)	0.85	1.65	1.96	1.99	2.16	2.12	2.36	1.81	4.30-5.80
hemoglobin (g/L)	29	51	61	65	69	68	76	56	130-175
platelets (×109/L)	92	61	109	134	148	144	46	18	125-350
monocytes(%)	17.0	18.5	16.4	9.2	10.0	10.5	34.0	30.0	3-10
prothrombin time(s)	15.3	/	12.9	/	12.6	/	13.7	/	9-13
the international normalized ratio	1.31	/	1.10	/	1.08	/	1.17	/	0.75-1.2
activated partial thromboplastin time(s)	25.6	/	26.5	/	24.2	/	24.4	/	20-40
thrombin time(s)	19.0	/	18.9	/	18.9	/	19.2	/	14-21
fibrinogen(g/L)	4.23	/	3.28	/	3.14	/	2.24	/	2-4
fibrin degradation products(mg/L)	4.88	/	3.59	/	3.42	/	8.84	/	0-5
D-dimer(mg/L)	2.03	/	1.34	/	0.89	/	2.56	/	0-0.5

“/”: not test.

## Discussion

Acute promyelocytic leukemia (APL) is a special type of acute myeloid leukemia (AML). Over 95% of cases exhibit the specific chromosomal translocation t (15;17) (q22; q21), forming the *PML:: RARα* fusion gene. The protein product of this fusion gene leads to blocked cell differentiation and insufficient apoptosis, which constitutes the primary molecular mechanism underlying APL pathogenesis (5, 6). The clinical manifestations of APL are dangerous, with a high risk of hemorrhage and thrombosis during disease onset and induction therapy, which can cause death. Its clinical and laboratory features differ significantly from other AML subtypes (7). APL with typical chromosomal translocations can be induced to differentiate into mature-like granulocytes by all-trans retinoic acid (8), demonstrating excellent responsiveness to ATRA therapy. In very rare cases (1–2%), APL may also present with variant translocations, including t(11;17) (11q23;q12)/*PLZF::RARα*, t(5;17) (5q35;q12)/*NPM::RARα*, t(11;17) (q13;q21)/*NUMA::RARα*, der(17)/*STAT5b::RAR*-RARα, t(17;17) (q24;q12)*/PRKAR1A::RARα*, t(4;17) (q12;q21)/*FIP1L1::RARα*, t (X;17) (p11;q21)/*BCOR::RARα*, t(2;17) (q32;q21)/*OBFC2A::RARα*,t(3;17) (q26;q21)/*TBLR1::RARα*, t(7;17) (q11;q21)/*GTF2I::RARα*, t(1;17) (q42;q21)/*RF2BP2::RARα*, and t(17;17) (17q21;q12)/*STAT3::RARα* (9–11)^10^. Here, we sort out and compare typical APL and non-M3 AML according to the latest WHO classification criteria from the perspectives of cytomorphology, immunology, cytogenetics and molecular biology (**Table 2**) (12–14). It can be seen from the table that typical APL has unique cytomorphological, immunological and cytogenetic characteristics. The most prominent morphological feature is the malignant proliferation of abnormal promyelocytes, whose morphology differs from that of normal promyelocytes. The characteristics are that the cell body size varies, often being round, nearly round or irregular in shape, the cell nucleus is slightly small, often biased to one side, the nuclear shape is irregular, and it is relatively easy to see nuclear distortion, folding, lobulation, butterfly-like and glute-like nuclei. The nuclear chromatin is loose, and obvious nucleolus can generally be seen. But some are covered by coarse particles and are not clear; The cytoplasm is abundant, containing a large number of purple-red Acanthopanax granules of varying sizes, mostly distributed at one end of the cytoplasm, around the nucleus or covering the nucleus. Some cells exhibit bilayer cytoplasm: the inner layer is filled with granules and located at the edge of the cell, while the outer layer has few or no granules and protrudes like pseudopods. Some cells contain numerous Auer rods, arranged in bundles or cross-striated patterns resembling faggots, hence termed “faggot cells”. In this case, peripheral blood and bone marrow smears revealed numerous “faggot cells”, with clear nucleolis, these abnormal cells exhibited strongly positive peroxidase (POX) and chloracetate esterase staining, which also contributed to the misdiagnosis in morphological assessment. However, in this case, the nuclei of the abnormal cells did not exhibit obvious nuclear twisting, lobulation, or folding. Most of them were relatively regular and round or nearly round. Only in this aspect, the morphology was different from typical abnormal promyelocytic cells. However, with the emergence of test results such as fluorescence *in situ* hybridization and whole transcriptome sequencing, all indicating the absence of RARα gene fusion or other rare variant fusions, the previous morphological inference was thereby overturned. To further clarify the significance of this special structure, relevant literature was reviewed. Currently, similar appearances of “faggot-like cells” have been reported in cases involving other non-myeloid cells. For instance, Bouanani N et al. reported their presence in plasma cells (15), Roldán Galiacho V et al. reported them in lymphocytes of chronic lymphocytic leukemia (16), and Gao Z et al. observed them in B-cell lymphoma cells (17). However, most of these Auer body-like inclusion substances tested negative for peroxidase(POX) staining, with some confirmed under electron microscopy to be lysosomal material. Others were speculated to be deposits resulting from the degradation of certain bacterial or mycoplasma products by autophagosomes (18). Meanwhile, some cases ultimately diagnosed as abnormal promyelocytes were misidentified as monoblasts or myeloblasts due to nuclear distortion, folding, and hypogranularity (19). However, similar cases like this one, where the cytoplasm is filled with typical “faggot cells” and the cytochemical staining results are also consistent, but only the nuclear karyotype is atypical, have rarely been reported. The typical phenotype of APL shows large SSC, absence of CD34 and HLA-DR expression, strong or uniform expression of CD33, homogeneous expression of CD13, CD9CD123, and CD64, and occasional expression of CD56, CD7, or CD2 (19, 20). About 10% of atypical APL cases (hypogranular variant) generally exhibit CD34 and/or HLA-DR expression, moderate SSC, and co-expression of CD2 or CD56 (21–23).Analysis of this case’s immunophenotype revealed: CD45dim+, CD117+, CD33+, CD64+, CD38+, CD15+, intracellular MPO+, partial HLA-DR+, partial CD123+, a small amount of CD13+, CD9-, CD34-,CD10-,CD14-,CD56-, CD4-, CD7-, CD19-, CD3-. Given this profile, APL could not be entirely ruled out, so the conclusion only stated that the immunophenotype was consistent with “acute myeloid leukemia(AML)” without specifying non-M3 subtype. Combining flow cytometry and morphological findings, we boldly hypothesize that the treatment regimen incorporating ATRA + ATO + azacitidine + venetoclax may also align with the progression of the disease. The patient achieved temporary remission after two chemotherapy cycles but later relapsed—an unexpected outcome. Due to the short survival period, the patient did not complete an ideal treatment course, nor was it possible to monitor efficacy further or investigate the mechanism and prognosis of the abnormal structures in the blasts, which is regrettable. Additionally, a rare fusion gene, *SLC66A2*::*CTDP1*, was detected. Reports on this fusion are scarce, with only a few documented cases in B-ALL. The SLC66A2 gene belongs to the solute carrier (SLC) family, potentially involved in transporting specific metabolites (e.g. amino acids, neurotransmitters), though its exact function remains unclear due to limited research. The CTDP1 gene encodes the C-terminal domain (CTD) phosphatase of RNA polymerase II, regulating transcriptional elongation and DNA damage repair, with links to neurodevelopment and cancer. The clinical significance of their fusion is currently unknown, with minimal literature available. Further clinical data are needed to elucidate these findings. Meanwhile, detected mutations in TET2, U2AF1, and other genes all indicate poor prognosis (24), and we speculate that this may be the reason for the patient’s further recurrence.

**Table 2:** Comparison table of classic APL (M3 type) and AML non-M3 type.

	Classic APL (M3 Type)	Non-M3 Type AML
Morphology	Abnormal promyelocytes ≥20% Cytoplasm : dense azurophilic granules **Nuclear shape: irregular(most)** reniform/folded/lobulated “Faggot-like “Auer bodies(most) MPO: strong positivity.	Myeloid blasts/immature cells ≥20% No abnormal promyelocytes Cytoplasm : azurophilic granules more or less **Nuclear shape** :normal(most), irregular(minority) Auer bodies (One to two or three)
Immunology	**Core markers: **CD34-, HLA-DR-; **Typical markers: ** CD33+ (strong), CD13+, CD117+, cytoplasmic MPO++; **Special markers: ** Hypogranular variant: CD34+/CD2+; CD56+	**Typical markers: ** CD34+, HLA-DR+ (except some mature subtypes) **Subtype: ** M0/M1 (CD13+, CD33+, CD117+, MPO+), M2 (same as M0/M1, may express CD15+), M4 (CD14+, CD64+, CD11b+), M5 (CD14+, CD64+, CD4+), M7 (CD41+, CD61+).
Cytogenetics	**Typical: ** t (15;17) (q22; q12) (>90%); **Rare:** t (11;17), t (5;17), etc.	**NO t (15;17)** **Normal karyotype :** 40%-50% **Common: ** t (8;21) (q22;q22) RUNX1::RUNX1T1 inv (16) (p13q22)/t (16;16) (p13.;q22) /CBFB::MYH11 t (9;11) (p22;q23) /MLLT:: KMT2A +8, -7, del(5q), etc.
Molecular Biology	**Typical:** PML::RARα fusion gene (95% ); **Concurrent mutations: ** FLT3-ITD, N-RAS, WT1, etc.	**NO PML**::**RARα fusion gene** **Fusion genes:** RUNX1::RUNX1T1, CBFB::MYH11, etc., **Gene mutation :** NPM1(30%), FLT3-ITD, CEBPA , TP53, etc.

## Conclusion

In summary, this case presents a diagnostic discussion of acute myeloid leukemia (non-M3type) with morphological features resembling “M3.” The emergence of such a peculiar phenomenon raises many questions. The presence of numerous “faggot-like Auer rods” in myeloblasts without definitive fusion gene occurrence prompts consideration—could this structural manifestation indicate a well-differentiated state? However, the patient’s survival period of merely over four months does not seem to suggest favorable prognosis. Do these unique structures influence treatment strategies? How to adjust therapeutic regimens poses another challenge for clinicians. We hope this case sharing may provide new insights for diagnosing similar clinical cases in the future. When strongly suspecting APL, ATRA treatment should be initiated immediately. If RARα fusion gene testing returns negative, further examinations remain necessary to exclude rare fusions. Cytomorphological diagnosis remains the basis for leukemia diagnosis, but other detection methods are still needed to supplement and improve it. Only by making a comprehensive judgment based on the results of MICM examination can we avoid falling into the misunderstandings of diagnosis and treatment.

## Data Availability

The raw sequencing data has been deposited in NCBI under the accession number: PRJNA1471434.

## References

[1] Shimony S Stahl M Stone RM . Acute myeloid leukemia: 2025 update on diagnosis and management. Am J Hematol. (2025) 100:860–91. doi: 10.1002/ajh.27625 39936576 PMC11966364

[2] Lo-Coco F Grimwade D Müller MC Saglio G Franke GN Voso MT . Acute promyelocytic leukemia: Current management and future perspectives. J Clin Oncol. (2013) 31:3137–48. doi: 10.1200/JCO.2012.45.8227 42148471

[3] Tallman MS Daver N Cortes J DiNardo CD Ritchie EK Stone RM . Acute promyelocytic leukemia: NCCN clinical practice guidelines in oncology (Version 2.2023). J Natl Compr Cancer Network. (2023) 21:875–87. doi: 10.6004/jnccn.2023.0049

[4] DiNardo CD Pratz KW Letai A Jonas BA Wei AH Arellano M . Safety and preliminary efficacy of venetoclax with decitabine or azacitidine in elderly patients with previously untreated acute myeloid leukaemia: a non-randomised, open-label, phase 1b study. Lancet Oncol. (2018) 19:216–28. doi: 10.1016/S1470-2045(18)30010-X 29339097

[5] Kakizuka A Miller RLJ Biggs WH Kadowaga TJ Kanno H Pandolfi PP . Chromosomal translocation t(15;17) in human acute promyelocytic leukemia fuses RAR alpha with a novel putative transcription factor, PML. Cell. (1991) 66:663–74. doi: 10.1016/0092-8674(91)90112-c 1652368

[6] Grignani F Ferrucci PF Testa U Talamo F Fagioli M Alcalay D . The acute promyelocytic leukemia-specific PML-RAR alpha fusion protein inhibits differentiation and promotes survival of myeloid precursor cells. Cell. (1993) 74:423–31. doi: 10.1016/0092-8674(93)80044-f 8394219

[7] Sabattini E Bacci F Sagramoso C Pileri SA . WHO classification of tumours of haematopoietic and lymphoid tissues in 2008: an overview. Pathologica. (2010) 102:83–7. doi: 10.1708/494.5814 21171509

[8] Powell BL Moser B Stock W Gallagher RE Willman CL Stone RM . Arsenic trioxide improves event-free and overall survival for adults with acute promyelocytic leukemia: North American Leukemia Intergroup Study C9710. Blood. (2010) 116:3751–7. doi: 10.1182/blood-2010-02-269621 20705755 PMC2981533

[9] Yan W Zhang G . Molecular characteristics and clinical significance of 12 fusion genes in acute promyelocytic leukemia: a systematic review. Acta Haematol. (2016) 136:1–15. doi: 10.1159/000444514 27089249

[10] Chen Y Li S Zhou C Li J Zhu J Wang YY . TBLR1 fuses to retinoid acid receptor α in a variant t(3;17)(q26;q21) translocation of acute promyelocytic leukemia. Blood. (2014) 124:936–45. doi: 10.1182/blood-2013-10-528596 24782508

[11] Yao L Wen L Wang N Zhou JF Chen P Wang M . Identification of novel recurrent STAT3-RARA fusions in acute promyelocytic leukemia lacking t(15;17)(q22;q12)/PML-RARA. Blood. (2018) 131:935–9. doi: 10.1182/blood-2017-09-807370 29237593

[12] Swerdlow SH Campo E Harris NL Pileri SA Jaffe ES Stein H . WHO Classification of Tumours of Haematopoietic and Lymphoid Tissues. 5th Edition. Lyon: International Agency for Research on Cancer (IARC (2022).

[13] Arber DA Orazi A Hasserjian R Borowitz MJ Le Beau MM Bloomfield CD . The 2022 international consensus classification of myeloid neoplasms and acute leukemia: A report of the international consensus classification (ICC) task force. Blood. (2022) 140:1204–49. doi: 10.1007/s00428-022-03430-4 36264379

[14] Döhner H Weisdorf DJ Bloomfield CD . Acute myeloid leukemia. N Engl J Med. (2022) 387:1039–51. doi: 10.1002/stem.5530150823 26376137

[15] Bouanani N Achour L Yahyaoui A Youssefi H . Auer rod-like inclusions in a patient with multiple myeloma. Balkan Med J. (2022) 39:446–7. doi: 10.4274/balkanmedj.galenos.2022.2022-8-69 36317725 PMC9667216

[16] Roldán Galiacho V Lobo Olmedo A Aranguren Del Castillo L Arzuaga-Mendez J García-Ruiz JC . Lymphocytes with auer rod-like inclusions in chronic lymphocytic leukemia. Hematol Transfus Cell Ther. (2022) 44:616–7. doi: 10.1016/j.htct.2021.06.018 34580044 PMC9605899

[17] Gao Z Cui F Liu M Guo Y Hu Y Shi M . Auer rod-like inclusions in the cytoplasm of B-cell lymphoma cells with bone marrow infiltration. Exp Hematol. (2019) 77:6–11. doi: 10.1016/j.exphem.2019.08.003 31442465

[18] Courville EL Shantzer L Vitzthum von Eckstaedt HC Guo L Hsi E Rogers HJ . Variant acute promyelocytic leukemia presenting without auer rods highlights the need for correlation with cytogenetic data in leukemia diagnosis. Lab Med. (2022) 53:95–9. doi: 10.1093/labmed/lmab051 34268555

[19] Yoshii M Ishida M Yoshida T Kato S Yamashita Y Horlike S . Clinicopathological features of acute promyelocytic leukemia: An experience in one institute emphasizing the morphological and immunophenotypic changes at the time of relapse. Int J Clin Exp Pathol. (2013) 6:2192–8. doi: 10.7150/ijcep.6679 PMC3796242 24133598

[20] Dunphy CH Tang W . The value of CD64 expression in distinguishing acute myeloid leukemia with monocytic differentiation from other subtypes of acute myeloid leukemia: A flow cytometric analysis of 64 cases. Arch Pathol Lab Med. (2007) 131:748–54. doi: 10.5858/2007-131-748-TVOCEI 17488160

[21] Albano F Mestice A Pannunzio A Rossi G Specchia G Musto P . The biological characteristics of CD34+ CD2+ adult acute promyelocytic leukemia and the CD34 CD2 hypergranular (M3) and microgranular (M3v) phenotypes. Haematologica. (2006) 91:311–6. doi: 10.3324/haematol.10694 16531253

[22] Di Noto R Mirabelli P Del Vecchio L . Flow cytometry analysis of acute promyelocytic leukemia: The power of 'surface hematology'. Leukemia. (2007) 21:4–8. doi: 10.1038/sj.leu.2404412 17167527

[23] Liu M Weng X Gong S Li Y Zhang J Wang Y . Flow cytometric analysis of CD64 expression pattern and density in the diagnosis of acute promyelocytic leukemia: A multi-center study in Shanghai, China. Oncotarget. (2017) 8:80625–37. doi: 10.18632/oncotarget.20814 29113330 PMC5655225

[24] Wang R Gao X Yu L . The prognostic impact of tet oncogene family member 2 mutations in patients with acute myeloid leukemia: A systematic-review and meta-analysis. BMC Cancer. (2019) 19:389. doi: 10.1186/s12885-019-5602-8 31023266 PMC6485112

